# Low dose oral ketamine treatment in chronic suicidality: An open-label pilot study

**DOI:** 10.1038/s41398-021-01230-z

**Published:** 2021-02-04

**Authors:** Adem T. Can, Daniel F. Hermens, Megan Dutton, Cyrana C. Gallay, Emma Jensen, Monique Jones, Jennifer Scherman, Denise A. Beaudequin, Cian Yang, Paul E. Schwenn, Jim Lagopoulos

**Affiliations:** Thompson Institute, University of the Sunshine Coast, Birtinya, Queensland Australia

**Keywords:** Depression, Scientific community

## Abstract

Recently, low-dose ketamine has been proposed as a rapid-acting treatment option for suicidality. The majority of studies to date have utilised intravenous (IV) ketamine, however, this route of administration has limitations. On the other hand, oral ketamine can be administered in a range of settings, which is important in treating suicidality, although studies as to safety and feasibility are lacking. *n* = 32 adults (aged 22–72 years; 53% female) with chronic suicidal thoughts participated in the Oral Ketamine Trial on Suicidality (OKTOS), an open-label trial of sub-anaesthetic doses of oral ketamine over 6 weeks. Participants commenced with 0.5 mg/kg of ketamine, which was titrated to a maximum 3.0 mg/kg. Follow-up assessments occurred at 4 weeks after the final dose. The primary outcome measure was the Beck Scale for Suicide Ideation (BSS) and secondary measures included scales for suicidality and depressive symptoms, and measures of functioning and well-being. Mean BSS scores significantly reduced from a high level of suicidal ideation at the pre-ketamine (week 0) timepoint to below the clinical threshold at the post-ketamine (week 6) timepoint. The proportion of participants that achieved clinical improvement within the first 6 weeks was 69%, whereas 50% achieved a significant improvement by the follow-up (week 10) timepoint. Six weeks of oral ketamine treatment in participants with chronic suicidality led to significant reduction in suicidal ideation. The response observed in this study is consistent with IV ketamine trials, suggesting that oral administration is a feasible and tolerable alternative treatment for chronic suicidality.

## Introduction

Suicide is deemed a significant public health concern worldwide. Although there has been an overall decline globally, suicide remains a leading cause of death, with an estimated one million deaths every year^1^. Vulnerability to suicide may involve many behavioural, environmental, genetic and neurobiological factors^2–6^. Distal risk factors include genetic loading, trait impulsivity and early traumatic life events, whereas proximal risk factors include psychological crisis, acute stress and a diagnosis of a mental illness^3,7,8^. It is estimated that 90% of suicides are associated with some form of psychiatric disorder, where 60% of suicides have been attributed to mood disorders^9–12^. In current clinical practice, suicidality interventions may involve treating depression, anxiety or addressing psychosocial stressors^13^. However, despite the application of these approaches, some individuals continue to experience ongoing suicidality. Chronic suicidality refers to experiencing suicidal ideation on a continuous or intermittent basis over a period of time, without the immediate risk implied by acute suicidality^13^.

Antidepressant medications are widely used globally to treat mood disorders and attendant suicidal ideation. Commencement of antidepressants can take weeks to have a positive effect and may actually increase suicidal thoughts and parasuicidal gestures, exposing individuals to periods of undertreated depression which can lead to attempts or death by suicide^14–16^. It is estimated that 30% of sufferers of depression fail to respond to at least two trials of antidepressants, leading to a diagnosis of treatment-resistant depression (TRD)^16^. Notably, traditional antidepressants do not directly affect the primary excitatory and inhibitory neurotransmitters (i.e. glutamate and GABA, respectively), and yet both are implicated in the pathophysiology of depression^17^. Recent literature suggests that the neuropathology of suicidality and depressive illnesses may differ, and suicidality may be independent from other symptoms in depression and anxiety. Therefore, successful treatment of mood disorders with traditional antidepressant therapy may have limited impact on suicidal ideation^18^.

Ketamine, a potent, non-competitive *N*-methyl-d-aspartate receptor antagonist, has been widely used in medicine for over 50 years as a general anaesthetic and short-acting analgesic agent. Ketamine enhances excitatory (glutamatergic) neurotransmission and also increases protein synthesis^19,20^. Thus, it has both transient (minutes-to-hours) and sustained (days-to-weeks) effects, whereby the initial postsynaptic glutamate activation leads to upregulation of neurotrophic signalling and increased protein synthesis, ultimately increasing synaptic formation and connectivity^21^. Animal studies have shown that ketamine-induced increased protein synthesis promotes an increase in functional synapse numbers in the prefrontal cortex, with a subsequent decrease in ‘despair-like’ behaviour^20,22^. In human trials, sub-anaesthetic, low-dose ketamine has been shown to promote rapid improvement in TRD^23–25^ and suicidal ideation^18,26–29^. While single or multiple doses of ketamine (over a period of weeks) has been shown to significantly reduce depressive and suicidal symptoms within hours, early relapse following discontinuation has also been reported^30^. Thus, identification of optimal dosing regimens and routes of administration, as well as strategies to maintain the antidepressant and antisuicidal response remains a key focus of research^30^.

To date, the majority of studies that have assessed the efficacy of ketamine on suicidality have utilised intravenous (IV) administration^31^. Three open-label single-dose studies evaluated the effect of IV ketamine on suicidality in patients with TRD^18,26,29^. All three studies found that suicidality reduced significantly, over several^18,29^ to 24 hours^26^ following ketamine infusion. Another open-label trial of IV ketamine in TRD reported a steady decrease in suicidal ideation over 6 consecutive weeks of infusions^28^. Three randomised controlled trials in patients with affective disorders and clinically significant suicidal ideation compared single, sub-anaesthetic doses of IV ketamine to the placebo drug, midazolam^27,32,33^. Across all three trials, patients who received a ketamine infusion showed significantly decreased suicidal ideation after 24 h compared to those who received midazolam^27,32,33^. In their systematic review of single-dose IV ketamine trials, Wilkinson et al.^34^ concluded that ketamine’s rapid and acute effects on suicidal ideation (with moderate-to-large effect sizes) were partially independent of its effects on mood.

IV administration of medications is costly and invasive, with potential complications such as phlebitis and infection and normally must be administered in a hospital setting. An oral form of ketamine that can be administered with ease, and potentially on a more frequent basis, is therefore an attractive option for the treatment of suicidal ideation. However, very few studies have explored the feasibility of low-dose oral ketamine in treating suicidality.

A proof-of-concept trial^35^ of 28-day, open-label, oral ketamine administered daily to patients receiving hospice care reported significant improvements in depressive and anxiety symptoms after 2 weeks, in over half the patients. The authors of this study noted that the observed response rate (57%) was similar to other studies that utilised IV ketamine treatment in patients with TRD. However, unlike other studies the response in their oral ketamine trial was protracted (i.e. taking weeks rather than minutes). Of note, Irwin et al.^35^ found that ‘suicide risk’ did not change with oral ketamine treatment. In a retrospective study of oral ketamine treatment in *n* = 22 patients with TRD, Al Shirawi et al.^36^ reported that a third (32%) of participants showed a clinically beneficial response in terms of depression scale ratings, whereas 45% were non-responders and 23% showed a mild worsening of depressive symptoms following ketamine treatment. This study was limited in terms of consistency with regards to dosing and regimen, whereby titration was continued every 3 days until patients showed either: (i) improved depressed mood; (ii) a lack of tolerability, without improvement in mood; or (iii) no improvement in mood despite reaching the maximum dose.

Given the results to date, there is a need to further investigate the potential benefit of oral ketamine in people with persistent suicidal ideation. However, there remain significant gaps in our knowledge about dosage levels, treatment protocols, and the feasibility and safety of oral ketamine’s short- and long-term use. The present study sought to address this issue by undertaking an open-label, flexible-dose, 6-week trial of oral ketamine with a 4-week (no ketamine) follow-up phase, to assess dosing regimen, safety/tolerability, and acute and prolonged responses in treating adults with chronic suicidality. The primary outcome measure was change in suicidality ratings and we hypothesised that oral ketamine would lead to a significant reduction in suicidality from baseline to post-ketamine visit.

## Subjects and methods

### Ethics approval and consent to participate

This study (A181101) was approved by the Bellberry Human Research Ethics Committee (HREC) and approval was subsequently ratified by the University of the Sunshine Coast HREC. The study was conducted in accordance with the ethical principles of the Declaration of Helsinki. All participants provided informed written consent. Compensation was provided in the form of fuel vouchers. The trial was registered with Australia and New Zealand Clinical Trials Registry (ACTRN12618001412224).

### Participants

Local general practitioners (GP) and psychiatrists were informed about the trial via phone calls and clinic visits. Approved advertising material was published on our website and provided to 11 local GP clinics and two private hospitals. Patients with chronic suicidality as the primary presenting complaint were referred to the study by their local GP, psychiatrists and psychologists. More specifically, ‘chronic suicidality’ was defined here as experiencing suicidal ideation of varying intensity on a continuous or intermittent basis over a period of months to years, with the ongoing likelihood of a person considering a future attempt. This was determined by the principal investigator and consultant psychiatrist (AC) who obtained a psychiatric history and assessed the mental state of participants including severity and chronicity of their suicidality. Adult participants (18+ years) were included in the study if they attained a Beck Scale for Suicide Ideation (BSS) score of ≥6 at screening. Exclusion criteria were: (i) history of psychosis and/or mania/hypomania; (ii) acute suicidality requiring urgent psychiatric intervention; and/or (iii) any of the following physical conditions: (a) uncontrolled/severe symptomatic cardiovascular disease states including: recent myocardial infarction (within prior 6 months); history of stroke; and hypertension (resting blood pressure >150/100); (b) history of intracranial mass, intracranial haemorrhage/stroke, cerebral trauma/traumatic brain injury or increased intracranial pressure (as assessed by referring general practitioner); (c) liver function test results out of normal range; (d) previous reaction to ketamine (as reported by referring general practitioner and participant); (e) pregnancy; (f) breastfeeding.

All participants were assessed as physically healthy, determined by physical examination, laboratory blood tests and medical history obtained by the principal investigator/consultant psychiatrist. During the treatment and follow-up phase, participants maintained their regular medication regimen as prescribed by their own health professionals. Concurrent psychopharmacological agents taken by participants included SSRI, SNRI and mood stabilisers. Table 1 summarises the DSM-5 diagnoses (primary, comorbidity) and concurrent psychotropic medications for each participant in the trial.

**Table 1:** DSM-5 diagnoses (*primary*, comorbidity) in addition to ‘chronic suicidality’ and corresponding psychotropic medications concurrently taken at the time of the ketamine trial.

ID	DSM-5 diagnosis	Psychotropic medication(s)
01	MDD, GAD	Quetiapine, Tranylcypromine, Chlorpromazine
02	MDD, GAD	Escitalopram
03	MDD	Sertraline
06	MDD, GAD	Quetiapine, Lithium, Imipramine, Chlorpromazine
07	MDD, GAD	Escitalopram, Propranolol
08	MDD, GAD	Sertraline
09	MDD, GAD	Mirtazapine
10	MDD, GAD	Fluoxetine
11	MDD	Duloxetine, Mirtazapine, Pregabalin
12	MDD, GAD, BPD	Vortioxetine, Valium, Fluoxetine
14	PTSD, MDD, GAD	Nil
15	MDD	Asenapine, Lithium, Sertraline, Bupropion
16	MDD	Amitriptyline
18	OCD, MDD	Nil
19	MDD, GAD	Duloxetine, Quetiapine
20	MDD, PTSD	Olanzapine, Mirtazapine, Duloxetine
21	MDD, BPD	Nil
22	MDD, PTSD	Desvenlafaxine, Mirtazapine, Lithium
23	MDD, BPD	Paroxetine
24	MDD	Nortriptyline, Quetiapine
25	MDD, GAD, BPD	Duloxetine, Lithium, Lorazepam
26	OCD, PTSD, MDD	Nil
27	GAD, MDD	Lithium, Clomipramine, Olanzapine, Zolpidem, Asenapine
28	MDD, GAD	Duloxetine, Amitriptyline
29	MDD	Nil
31	MDD, GAD	Sertraline, Quetiapine
33	MDD, GAD, BPD, SUD*	Fluoxetine, Asenapine
34	MDD, GAD, PD	Escitalopram
35	MDD	Fluoxetine
38	MDD, GAD	Duloxetine, Amitriptyline, Pregabalin
39	MDD, PND	Desvenlafaxine
40	MDD, PTSD	Oxazepam

*BPD* Borderline Personality Disorder, *GAD* Generalised Anxiety Disorder, *MDD* Major Depressive Disorder, *OCD* Obsessive Compulsive Disorder, *PD* Panic Disorder, *PND* Post-natal Depression, *PTSD* Post Traumatic Stress Disorder, SUD* = Substance Use Disorder (in Remission).

### Study design and treatment

This open-label trial of oral ketamine was conducted at the Thompson Institute between August 2018 and November 2019. The intervention consisted of 6 weeks of flexible-dose treatment with oral (racemic) ketamine and a 4-week follow-up phase (i.e., with no ketamine), to assess dosing regimen, the potential acute and prolonged responses and safety/tolerability of weekly oral ketamine administration for the treatment of chronic suicidality. Participants underwent baseline assessment comprising physical examination, urinalysis, laboratory blood testing and medical history within 14-days prior to commencement of ketamine treatment. Baseline assessment also included administration of standardised rating scales, as well as magnetic resonance imaging (MRI) and electroencephalogram (EEG) assessments (data not reported here).

**Fig. 1:**
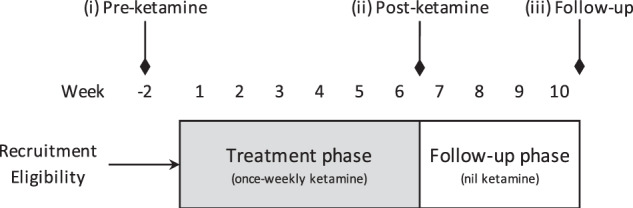
Oral Ketamine Trial on Suicidality (OKTOS) design. Following recruitment and determination of eligibility, OKTOS participants underwent (i) baseline (‘Pre-ketamine’) assessments within 14 days prior to commencing treatment. Treatment phase involved once-weekly doses of oral ketamine; this was followed by 4 weeks of nil ketamine. Two major endpoints: (ii) ‘Post-ketamine’ (week 6); and (iii) ‘Follow-up’ (week 10).

Participants received six oral, sub-anaesthetic doses of ketamine over 6 weeks, at one dose per week. The initial dose of ketamine was 0.5 mg/kg, administered as a liquid in fruit juice by the consultant psychiatrist. Dose amounts were titrated up by 0.2–0.5 mg/kg or down by 0.2–0.7 mg/kg at each treatment, depending on patient tolerance, with a maximum dose of 3.0 mg/kg at the sixth treatment. Tolerance to ketamine was determined by the presence or absence of side effects as measured by tolerability and safety rating scales and urinalysis. Participants were supervised immediately post treatment (for at least 90 min) and all were seen by the consultant psychiatrist before leaving the study premises. Participants were advised to avoid activities that required alertness (e.g. drive a vehicle, attend their workplace, enter legal agreements) for 12 h post treatment. Furthermore, they were asked to arrange for suitable transport to and from the site on the day of the treatment. During the trial, participants were assessed via nine study visits and via eleven phone assessments between visits. Figure 1 depicts the overall trial design with the three major timepoints: (i) ‘baseline’ (i.e. −2 weeks prior to treatment; termed: “pre-ketamine”), with visits once per week during treatment weeks 1–6 and then at two additional visits: (ii) “post-ketamine” and (iii) “follow-up” (i.e. 1–7 days and 28–32 days after the final treatment, respectively). Phone assessments took place twice a week after treatments 1–5 and once after treatment 6 to check for adverse side effects.

Vital signs (heart rate, blood pressure, oxygen saturation level and temperature) were recorded at the pre-ketamine visit as well as three times at the treatment visit (i.e. prior to ketamine administration and at 30 mins and 60 mins post-treatment). Blood samples were taken at the four main timepoints: i.e. pre-ketamine, at the 3rd weekly treatment, post-ketamine and follow-up. Serum haematological and biochemical parameters monitored included cholesterol, morning cortisol, brain-derived neurotropic factor, thyroid function, full blood count, liver function, urea and electrolytes. Urinalysis was performed at the pre-ketamine visit as well as prior to all treatments to screen for possible cystitis. Urine pregnancy screening was undertaken for females of childbearing age at the pre- and post-ketamine timepoints.

The following rating scales were undertaken weekly as well as at the pre-ketamine and follow-up timepoints by participants, to assess primary and secondary trial outcomes: (i) Beck Scale for Suicide Ideation (BSS), a 21-item clinical research instrument designed to quantify and assess suicidal intention^37,38^; (ii) Suicidal Ideation Attributes Scale (SIDAS), a five-item scale assessing frequency, controllability, closeness to attempt, distress and interference with daily activities of suicidal ideation over the past month^39^; (iii) Montgomery-Asberg Depression Rating Scale (MADRS) was used to measure the severity of depressive symptoms^40^; (iv) Social and Occupational Functioning Assessment Scale (SOFAS), a clinician-rated single-item scale used to indicate an individual’s level of social and occupational functioning along a continuum ranging from optimum functioning to functional impairment, independent of symptoms^41^; (v) World Health Organization Well-Being Index (WHO-5) is a short (5-item) global rating scale measuring subjective well-being^42^.

### Outcome measures

The primary outcome measure for the study was a reduction in suicidality with ketamine treatment, as determined by the BSS. A ‘response’ was defined by: (i) ⩾50% improvement in BSS score from the pre-ketamine to the post-ketamine visit; or (ii) BSS score ⩽6 at the post-ketamine visit. A ‘prolonged response’ was defined by: (i) ⩾50% improvement in BSS score from pre-ketamine to the follow-up visit; or (ii) BSS score ⩽6 at the follow-up visit. Secondary outcome measures included pre-ketamine to follow-up changes in SIDAS, MADRS, WHO-5 and SOFAS scores. Furthermore, ‘prolonged antidepressant response’ was defined by ⩾50% improvement in the MADRS score from pre-ketamine to the follow-up visit.

### Safety and tolerability

The following safety and tolerability measures were undertaken 60 min after each ketamine dose: (i) the Patient Rated Inventory of Side Effects (PRISE)^43^ was used to identify side effects and evaluate their tolerability; (ii) the Frequency, Intensity, and Burden of Side Effects Rating Scale (FIBSER)^44^ was used as global ratings of all side effects; (iii) the Clinician Administered Dissociative States Scale (CADSS)^45^ was used to check for present-state dissociative symptoms; the Young Mania Rating Scale (YMRS)^46^ was utilised to check for elevated mood (i.e. mania); and (iv) the Brief Psychiatric Rating Scale (BPRS)^47^ is general measure of psychiatric symptoms such as depression, anxiety, hallucinations and unusual behaviour. Furthermore, CADSS, YMRS and BPRS items relating to positive symptoms were closely monitored for potential side effects. Twice weekly phone calls by study mental health nurses were also conducted for additional safety monitoring.

### Statistical analyses

All statistical analyses were completed using SPSS version 24.0 (IBM Corp 2016). To test the primary outcome, a repeated measures ANOVA was conducted with time as the within-subjects factor (three levels: pre-ketamine, post-ketamine and follow-up timepoints) and BSS as the dependent variable. Pairwise comparisons of the timepoints were performed with Bonferroni corrections. Cohen’s *d* effect sizes were also calculated to further describe differences between timepoints. Sphericity assumption was confirmed via Mauchly’s test. For secondary outcomes (SIDAS, MADRS, SOFAS and WHO-5), repeated measures ANOVAs were conducted with two levels of time (pre-ketamine and follow-up) as a within-subjects factor. For all tests, significance thresholds were set at *p* = 0.05.

## Results

Of 64 participants screened, 40 (63%) met the inclusion criteria for the study. Of these, 5 were withdrawn from the study due to clinical concerns, and 3 elected not to continue with treatment. Thus, a total of *n* = 32 participants took part in the trial. While *n* = 32 participants completed 6 weeks of treatment and underwent the ‘post-ketamine’ timepoint assessments, *n* = 30 returned for the follow-up assessment (i.e. *n* = 2 participants were lost to follow-up).

Participants (17 females, 15 males) had a mean age of 45.7 ± 14.2 years and mean weight at first treatment of 91.1 ± 24.8 kg. In addition to ‘chronic suicidality’ being the primary presenting clinical feature, all participants met DSM-5 criteria for major depressive disorder (MDD). As summarised in Table 1, *n* = 28 (87%) of participants had MDD as their primary DSM-5 diagnosis, with *n* = 2 having OCD, *n* = 1 having PTSD and *n* = 1 having GAD as the primary diagnosis (the latter all had MDD as a comorbid diagnosis). Furthermore, *n* = 5 (16%) were not taking any concurrent medications at the commencement of the ketamine trial (see Table 1 for details of concurrent medications). Two-thirds (*n* = 21) were unemployed or not in labour force. Thirteen participants (40%) were married or in a de facto relationship at the time of the trial, two-thirds had tertiary level education and all but one participant (97%) had secure accommodation.

### Primary outcome measure

**Fig. 2:**
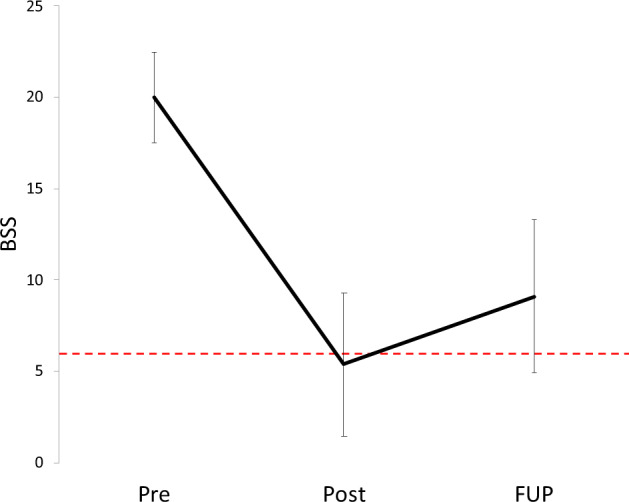
Mean Beck Scale for Suicidal Ideation (BSS) scores across three key timepoints. “Pre” = pre-ketamine, “Post” = post-ketamine, “FUP” = follow-up; dashed red line denotes cut-off (BSS ⩽ 6) for clinically significant suicidal ideation; error bars denote 99% CIs.

The mean BSS score was significantly reduced at both post-ketamine (5.6 ± 7.6, *n* = 32; range = 0–22) and follow-up (9.1 ± 8.2, *n* = 30; range = 0–25) timepoints (see Fig. 2) when compared to the pre-ketamine timepoint (20.0 ± 4.7, *n* = 32; range = 12–30). This was confirmed by omnibus ANOVA (main effect of time; *p* < 0.001) and significant pairwise comparisons between pre-ketamine and post ketamine (*p* < 0.001; *d* = 2.04) and follow-up (*p* < 0.001; *d* = 1.54) timepoints, respectively. Also, there was a significant increase in BSS score from post-ketamine to follow-up (pairwise comparison, *p* < 0.05; *d* = −0.42). Thus, at the group level, the BSS score reduced to be at or below the clinical threshold score of 6 at the post-ketamine timepoint. Furthermore, the mean follow-up BSS remained below 50% of the mean pre-ketamine BSS score (despite the significant increase from the post-ketamine level).

The proportion of BSS responders was 69%, while 50% were found to be BSS prolonged responders. The responder criterion of BSS ⩽6 was met by 95% at post-ketamine (all of whom also showed a ⩾50% improvement in BSS score) and 80% at follow-up. Of note, *n* = 13 trial participants were BSS responders at both post-ketamine and follow-up timepoints.

### Secondary outcome measures

The secondary outcome measures, recorded at the pre-ketamine and follow-up timepoints only, all showed significant changes (see Table 2). Specifically, the SIDAS scores reduced from 26.7 to 17.2 (*p* < 0.001), representing a categorical change from ‘high suicidal ideation’ to ‘low suicidal ideation’. The MADRS scores decreased from 38.6 (severe) to 15.9 (mild), SOFAS scores improved from 57.3 (moderate difficulty) to 69.3 (some difficulty) and WHO-5 well-being levels increased from 17.6% to 33.6% (despite the increase, the group remain in the ‘poor well-being’ range; i.e. <50%). The proportion of prolonged antidepressant (as determined by MADRS) responders was 63%. Of note, *n* = 13 of these responders were also BSS prolonged responders (i.e. *n* = 6 MADRS responders were not prolonged BSS responders).

**Table 2:** Mean scores (±standard deviation) for secondary outcome measures across pre-ketamine and follow-up timepoints.

	Pre-ketamine (*n* = 30)	Follow-up (*n* = 30)	Sig. test [*p*], Effect size (*d*)
SIDAS total	26.7 ± 8.7	17.2 ± 8.2	*F* (1, 29) = 21.9 [0.000], *d* = 0.85
MADRS total	38.6 ± 7.7	15.9 ± 11.8	*F* (1, 29) = 84.0 [0.000], *d* = 1.67
SOFAS	57.3 ± 9.1	69.3 ± 19.3	*F* (1, 29) = 12.9 [0.001], *d* = −0.62
WHO-5	17.6 ± 17.2	33.6 ± 29.4	*F* (1, 29) = 11.6 [0.002], *d* = −0.66

Legend: SIDAS, MADRS, SOFAS, WHO-5.

*SIDAS* Suicidal Ideation Attributes Scale, *MADRS* Montgomery-Åsberg Depression Rating Scale, *SOFAS* Social and Occupational Functioning Assessment Scale, *WHO-5* World Health Organisation-Five Well-Being Index.

### Adverse events and side effects

No serious adverse events were recorded during the study. Transient changes in vital signs were observed. No significant changes in heart rate, respiratory rate or oxygen saturation were observed. Maximum elevations in systolic blood pressure occurred at 30 mins post-ketamine. The mean increase in systolic blood pressure from the pre-ketamine time-point to 30 mins post-ketamine was 3.6 mmHg. Maximum increases in diastolic blood pressure also occurred at 30 mins post-ketamine. The mean increase in diastolic blood pressure from the pre-ketamine time-point to 30 mins post-ketamine was 4.7 mmHg. The treatment was generally well tolerated with no participants withdrawing from the trial due to ketamine side effects. The most frequent side effects throughout the 6 weeks of ketamine treatment were decreased energy and fatigue, followed by anxiety, poor concentration, restlessness, general malaise, dry mouth, dizziness and tremors. The general pattern across all symptoms was that the number of participants who rated these as being tolerable or distressing was largest in week 1 and tended to decrease across weeks 2–6, furthermore, the proportion of distressing symptoms decreased to low numbers (~22% for fatigue; and <10% across all other symptoms) by week 6 (see Table 3). In terms of overall intensity and burden of these symptoms, the majority of participants (~94%; *n* = 30/32) rated their symptoms to be mild. Similarly, in terms of burden the majority (*n* = 31/32) rated overall symptoms to be mild across the 6 weeks. In terms of dissociation, no participants rated any of the CADSS items above 2 (i.e. moderate) across the 6 weeks of treatment. The most commonly endorsed CADSS item at the ‘moderate’ level was ‘Do things seem to be moving in slow motion?’ (7 participants, no more than twice), followed by ‘Do things seem to be unreal to you, as if you are in a dream?’ (6 participants, no more than once), then ‘Does your sense of your own body feel changed…’ (5 participants, no more than once), then ‘Do things seem very real, as if there is a special sense of clarity?’ (4 participants, no more than once), and ‘Do you have some experience that separates you from what is happening…’ (2 participants, no more than once). Additionally, none of the 8 BPRS positive-symptom items exceeded a rating of 3 (mild) and no participants had a YMRS total score above 6 (well below the threshold for mania, i.e., 12 or more). Of note, only two participants had a total YMRS score of 6. For one of these, this was due to elevated mood and increase speech rate/amount following their week 3 dose, for the other this was also due to increase speech rate/amount (weeks 4-6), as well as irritability (week 4 only). Of note, across all weeks no participants displayed any of the YMRS positive (i.e. thought disorder, grandiosity/delusions) or disruptive/aggressive symptoms.

**Table 3:** Patient Rated Inventory of Side Effects (PRISE) counts of symptoms (rated tolerable or distressing) 60 mins post oral ketamine administration by treatment week (*n* = 32).

Treatment week	1	2	3	4	5	6
	*N*_tolerable_/*N*_distressing_					
Decreased energy	17/14	23/7	21/7	21/8	18/7	23/3
Fatigue	18/12	20/11	18/10	20/8	18/7	19/7
Anxiety	11/18	21/7	20/9	23/3	15/9	21/3
Poor concentration	16/13	22/6	19/7	18/7	20/4	19/2
Restlessness	19/5	15/3	17/4	14/3	15/3	16/1
General malaise discomfort	14/7	18/3	14/2	10/3	13/2	12/2
Dry mouth	17/1	15/3	18/2	19/2	15/2	18/1
Headache	16/1	19/2	15/3	14/1	15/1	12/3
Dizziness on standing	15/1	12/0	11/0	13/0	11/1	11/0
Tremors	13/2	10/1	9/2	11/1	9/1	9/0
Dry skin	13/0	12/0	12/0	14/0	13/0	13/0
Dizziness	13/0	10/0	13/0	9/0	9/0	11/0
Ringing in ears	10/2	8/0	7/1	5/1	5/1	7/1
Palpitation	10/1	12/0	9/1	8/0	8/0	6/0
Itching	10/0	8/0	8/0	8/0	7/0	5/1
Frequent urination	9/0	7/1	8/0	10/0	7/1	11/1
Blurred vision	8/0	3/0	5/0	6/0	6/0	7/0
Increased perspiration	8/0	4/0	4/0	4/0	3/0	3/1
Poor coordination	7/1	3/1	7/0	7/1	10/1	7/1
Rash	7/0	4/0	5/0	5/0	5/0	4/0
Nausea vomiting	6/0	7/1	7/1	10/0	6/1	11/0
Chest pain	4/1	3/0	1/0	2/0	2/0	5/0
Diarrhoea	4/0	6/1	6/0	4/0	5/0	5/0
Other	3/1	3/2	0/0	2/1	3/0	1/1
Constipation	3/0	7/0	5/0	7/0	9/0	9/0
Difficulty urinating	2/0	2/0	2/0	2/0	2/0	1/0
Painful urination	1/0	1/0	1/0	1/0	2/0	2/0

*N*_tolerable_/*N*_distressing_ is the number of individuals that reported the symptom to be tolerable or distressing.

## Discussion

To the best of our knowledge, the current study is the first to explore the feasibility, safety and tolerability of oral ketamine on chronic suicidality in patients who presented with a range of psychiatric conditions including mood, anxiety, and personality disorders. In this pilot study, 6 weeks of oral ketamine treatment for chronic suicidality led to a significant decrease in the primary outcome measure (BSS) from pre-ketamine to post-ketamine and from pre-ketamine to a follow-up period during which ketamine had ceased for 4 weeks following the post-ketamine timepoint. A slight increase in BSS scores was observed between the post-ketamine and follow-up timepoints; however, the sample mean score at follow-up was still representative of a >50% improvement in BSS from the pre-ketamine level. In terms of clinical significance, the effect size for change in BSS score from pre-ketamine to post-ketamine timepoints was very large (Cohen’s *d* > 2.0) and remained very large (*d* > 1.5) at follow-up (i.e. despite the increase in BSS score from post-ketamine to follow-up). The other suicidality and depression scales reflected the same overall pattern, for both self-reported and clinician-rated scales. On closer inspection, these scales revealed some important details. The clinician-rated measure for depressive symptoms (MADRS) was in the ‘mild’ range at follow-up. Furthermore, for the self-reported measure of suicidal ideation (i.e. the SIDAS) the mean score was in the low risk category.

In terms of the functional and well-being measures (i.e. SOFAS and WHO-5), the improvements (at follow-up) were incremental, which may be due to the relatively brief period of time (10 weeks) for these constructs to change substantially. Overall, oral ketamine led to significant short-term and prolonged improvements in suicidal ideation, affective symptoms, well-being and socio-occupational functioning in this sample of adults with a history of chronic suicidality and MDD. Furthermore, this trial demonstrated feasibility, beyond the clinically significant reduction in suicidality and overall tolerability, by exceeding the target enrolment (of *n* = 25) within the funded period of 24 months (as per the trial registry).

There were some important findings in terms of the proportions of participants that responded to oral ketamine treatment. More than two-thirds (69%) of participants had an antisuicidal response (i.e. at week 6), and half (50%) showed a prolonged antisuicidal response (week 10). These response levels are consistent with those seen in trials using IV ketamine^27,28,48^, suggesting that oral ketamine is a viable form of treatment for chronic suicidality. Interestingly, a similar proportion of individuals (63%) were (prolonged) antidepressant responders, however a third of these were not categorised as prolonged antisuicidal responders (i.e. *n* = 6 individuals had ⩾50% improvement in the MADRS but not in BSS at follow-up), suggesting there may be individual differences in the way suicidal versus broader depressive symptoms may be improved by oral ketamine. This is consistent with findings that the reduction of suicidal ideation after ketamine treatment is variable in terms of the extent and duration^49^. Furthermore, the differences observed in responder-type are also consistent with the notion that ketamine’s effects on suicidality may be partially independent of its effects on other depressive symptoms^34^.

A distinct and contemporary feature of this study was that the treatment protocol also assessed specific dosing, titration method, and interval timeframes of oral ketamine. Our approach was conservative in terms of the maximum as well as the frequency of dose. The maximum oral ketamine dosage was capped at 3 mg/kg which is in contrast to previous studies that have reported dosages of up to 7 mg/kg^50^. Moreover, in direct contrast to our study where oral ketamine was administered once per week, previous studies have reported more frequent doses. Utilising such a conservative approach, we found that oral ketamine was relatively well tolerated as reflected by the fact that it was associated with only temporary side effects such as altered sensorium, dissociative experiences, sedation, dizziness and nausea immediately after the treatment. Notably, all of these side effects resolved by the time all participants were discharged. Despite this, based on current knowledge, oral ketamine should be taken under medical supervision until dosing and associated tolerability are well established in future studies.

The current study has several limitations that require mention. Firstly, it was an open-label study with no placebo or control group. Furthermore, given the design (that included 20 contact points) it is important to consider the possibility that these findings may in part be due to other factors such as non-specific psychological and/or expectancy effects. Thus, future studies of oral ketamine in chronic suicidality utilising randomised controlled designs are needed. Additionally, ketamine was administered as an augmentation therapy, meaning that participants were allowed to remain on their usual treatment. Therefore, we are not able to disentangle the contribution of the participant’s ongoing medications when reporting on participant improvement in well-being. In this respect it is difficult to exclude any combined and/or synergic effects between ketamine and other psychopharmacological agents or psychological interventions. Another limitation was that we did not specify a minimum duration for ‘chronic suicidality’, however, all participants in this trial experienced suicidality for at least 6 months. Future studies may address this by determining an a priori definition that specifies the duration of suicidality. This study sought to explore the feasibility, safety and tolerability of oral ketamine treatment in adults with chronic suicidality. While any determination of efficacy is limited by a lack of a control arm (and randomisation), the overall findings, given the inclusion criteria, flexible dosing (and mode of delivery) and acceptance of concurrent psychopharmacological agents suggest an overall feasibility and tolerability of oral ketamine in this population thereby providing a foundation for subsequent efficacy and comparative effectiveness trials.

The present study has added further evidence to the rapidly increasing ketamine literature regarding the feasibility of glutamatergic based antisuicidal agents. It highlights the importance of the glutamatergic system in treating suicidality (and depression) in contrast to current antidepressants, which primarily targets monoaminergic neurotransmitters. Larger randomised control studies are needed to examine whether the antisuicidal effect of ketamine can be separated from its antidepressant effects. Also, further studies are needed to explore the impact of ketamine on suicidal behaviour and suicidal attempts.
